# Construction of smart older adults care service model driven by primary health care

**DOI:** 10.3389/fpubh.2023.1157758

**Published:** 2023-04-17

**Authors:** Lechuan Zhang, Xiaoyan Xu

**Affiliations:** ^1^Shanghai Lixin University of Accounting and Finance, Shanghai, China; ^2^Shanghai Institute of Technology, Shanghai, China

**Keywords:** smart senior care service model, traditional older adults care service model, public health, primary medical and health care, pension service quality

## Abstract

In recent years, with the arrival of an overall aging population, how to provide for the elderly has become the focus of government departments and society. The traditional elderly care service model has problems such as backward construction of information platforms, low quality of elderly care services, and “digital divide”. For this reason, based on grassroots medical and health care, this paper improves the quality of elderly care services by establishing a smart elderly care service model. Through experiments, it can be found that compared with the traditional elderly care service model, the intelligent elderly care service model has an absolute advantage in the identification of nursing data. The recognition accuracy rate of the smart elderly care service model for all kinds of daily care data is above 94%, while the recognition accuracy rate of the traditional elderly care service model is below 90%. Therefore, it is of great significance to study the smart elderly care service model driven by primary medical care and health.

## 1. Introduction

A variety of pension problems arising from the aging process of the population continue to affect China’s social and economic progress. As urbanization continues to accelerate, the number of older adult people continues to grow, which has led to an increasing demand for older adults care services. This has added relatively high pressure on government departments and the pressure continues to rise (1). At this stage, practical research on smart senior care service mode has begun in many places. However, from the current actual situation, there are still some problems in the development of the smart senior care model, such as restricted by the structural imbalance of “supply and demand dislocation” of China’s older adults care services, the shortage of smart older adults care products and the insufficient supply power of the community smart older adults care products market. This requires improving the level of resource optimization and configuration of the smart senior care service platform to provide professional resources for the smart senior care service model. With the popularity of the concept of a new smart city, information technology and communication technology for public services based on a variety of technologies emerge endlessly, such as public service information technology based on high-speed broadband transmission, Internet of Things, mobile Internet, big data, etc., increasing and expanding the connotation and extension of public services. The “smart senior care” service industry is one of the common industries for the implementation of new smart cities (2, 3). Smart pension can further reduce the information asymmetry between the supply and demand sides. Various resources of senior care services are integrated to optimize the efficiency of distribution and supply of service resources and provide direction for the growth of senior care services.

With the aggravation of aging, nowadays, people pay more and more attention to senior care services. How to better build a smart older adults care model is a topic that many experts and scholars are studying, and research literature is also emerging in endlessly. Jin Xinyu proposed an organic connection with the three pension modes of institutional older adults care, community older adults care and home-based older adults care by taking diagnosis and treatment of large hospitals as the leading role and using the Internet. He integrated the new technology of artificial intelligence cloud diagnosis, treatment, and rehabilitation of geriatrics to establish the older adults care model of “medical and nursing wisdom linkage” (4). Liu Lijuan used a decision tree classification method to classify retirement data in order to more accurately predict the retirement intentions of older adult people in the community. By comparing the information gain and information gain rate of the sample data features, he determined the feature ranking and built a decision tree model (5). Shi Lifang explored the opportunities for the growth of smart older adults services in both urban and rural areas, breaking the urban–rural dichotomy and encouraging urban and rural areas to enjoy the benefits of reform and development (6). Li Chunsheng suggested an effective multidimensional attentional convolutional neural network model to analyze customer review texts and predict the quality of senior care services (7). There are a variety of research methods used by researchers in smart older adults care services, but little consideration is given to the use of grassroots medical and health drivers to study them.

Some researchers have other views on the research of smart older adults care service model. Wen Zhi proposed a comprehensive hybrid aggregation method with personalized quantifiers to select older adults service providers, where the personalized quantifiers used cubic spline interpolation to determine the location weights of the criteria (8). Sun Weipin believed that a sound social old-age security system could not only meet the needs of the older adults, but also make young people worry free. He provided residents with a higher level of pension insurance by studying the operation effect of the system (9). Jung Soo-Yong took the scientists and technicians of small and medium-sized enterprises who had joined the older adults care service as the research object to investigate the impact on the intention to continue to join the older adults care service. He found the difference between safe and profitable participants in older adults care services. He collected data through questionnaire survey and conducted empirical analysis (10). Wei Yuanting considered non-profit organizations as a potential social capital and an essential force to complement the work of older adults care. He explored the path through social surveys and studied the participation of non-profit organizations in social older adults care services (11). Many scholars have studied the older adults care service. Therefore, this study is meaningful.

How to truly understand the new definition of intelligent older adults care should not only stay on the surface, but also emphasize its artificial intelligence and high-tech services. The wisdom of smart senior care is penetrated into all aspects of personalized older adults care, which requires close integration of smart technology and older adults care services. Primary health care drives are applied to various segments, such as older adults care products, services, management, operation, and supervision, to enrich the content of older adults services, thus building a synergistic smart older adults service model.

## 2. Smart older adults care service related theories

### 2.1. Smart older adults care

**Figure 1:**
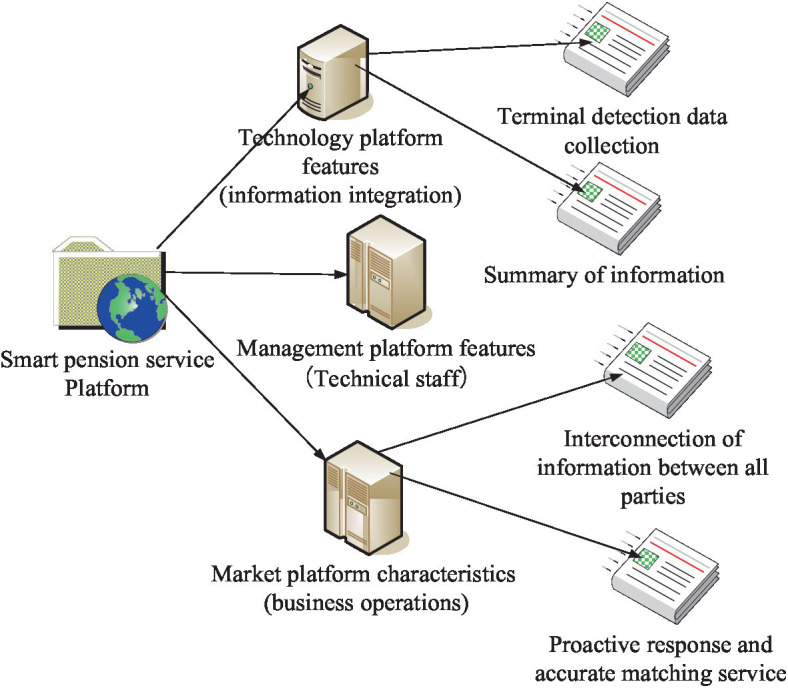
Features of the intelligent senior care service platform.

Based on the information technology such as Internet technology and wireless sensor network, the smart older adults system can help medical personnel and children to remotely monitor the daily life condition of the older adults and precisely locate their geographical location and the number of various health indicators, which can effectively prevent the occurrence of tragedies (12). The key to smart aging is to link the older adults with home care, nursing and other older adults service practitioners with the help of cutting-edge technology management methods to form a special unity of older adults services and an organic whole that can provide the older adults with interconnected, intelligent, more efficient and convenient older adults care services. It can, in turn, provide professional services for the living condition and recovery of the older adults, so that they can live a peaceful, physically and mentally healthy and happy daily life in their old age (13). It is to utilize a variety of modern information technology means, such as the Internet, the Internet of Things, social networking, big data, cloud computing, wireless sensor network systems, and other modern information technology means, to create an information platform to closely integrate with homes, communities and senior care service institutions. With the requirements of the older adults as the starting point, relevant data are automatically detected, analyzed, forewarned, communicated, responded, accurately positioned, and actively processed in real time to support and meet the needs of the older adults to the greatest extent. The features of the intelligent senior care service platform are shown in Figure 1.

Under the integration and guidance of high-tech information technology, older adults care services have become more and more intelligent, detailed and diversified (14). Nowadays, smart older adults care services focus on many aspects. Under the premise of enhancing the quality of life of the older adults, the accumulated experience and wisdom of the older adults should also be brought into full play, so that the two can be integrated with each other.

**Figure 2:**
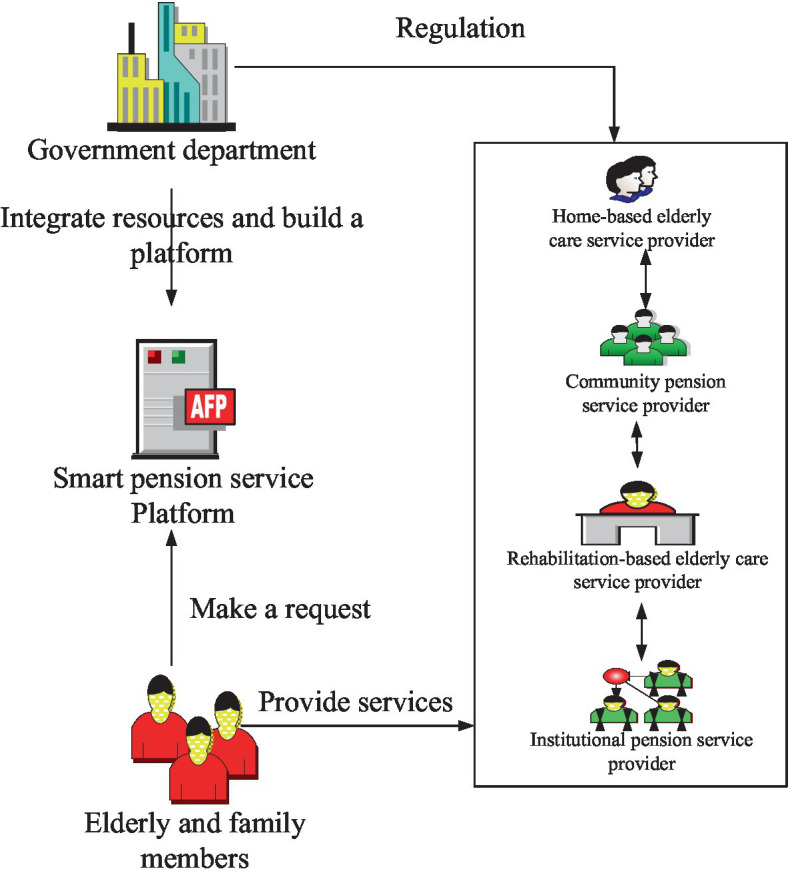
Basic service mode of smart older adults care.

Smart senior care service is a service obtained from the transformation of information technology. Its service mode is shown in Figure 2. Various emerging Internet information technologies are used to construct a smart senior care service platform and integrate senior care service resources including regional senior care service management suppliers, designated medical institutions, senior care service providers, and older adults family members. It can quickly respond to the service requirements of the older adults, and complete the rational connection between supply and demand, thus enhancing the service provision capability.

### 2.2. Smart older adults care service platform model

The concept of “platform” may have different understandings from different perspectives of the subject field. In the field of social economics, the concept of “platform” is defined from the perspective of industry exchange, and it is believed that “platform space” is a space or place to facilitate mutual or multi-faceted transactions, which can exist in the real world, such as large shopping malls. It can also exist in the Internet space, such as e-commerce platforms. The field of enterprise management defines the platform from market competition and enterprise relationship. The essence of the platform is to integrate resources to form a new system to improve its core competitiveness. Science and engineering disciplines emphasize the technical features of the “platform.” For example, the basic software platform is defined as an integrated system composed of operating systems, middleware, databases, security products and office suites based on the protocol rules between components.

**Figure 3:**
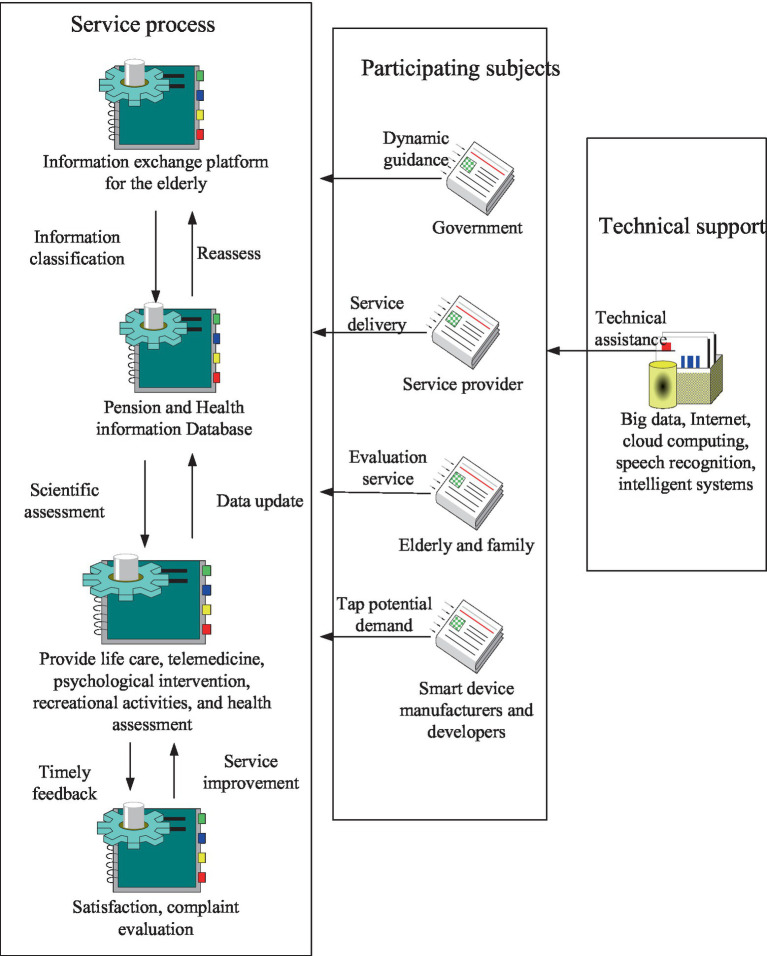
Framework of smart older adults care service model.

In terms of smart senior care service model and existing research on smart senior care, the key of smart senior care service model includes two parts. The first is the platform itself, and the second is the smart older adults care service supply mode relying on the platform. As an intermediary between supply and demand, the smart senior service platform realizes its value based on active response and providing intelligent matching services to give reliable business management services. The architecture design of the platform itself reflects the technical characteristics of the platform. Scientific and reasonable system architecture is the material condition to promote the operation of intelligent senior care service platform. The existing scientific research in the field of business operation mode defines the platform business service mode as gathering a number of significantly different but interdependent target customers. The service provision mode of the smart older adults care service platform is in line with the characteristics of the platform’s business operation mode to a certain extent. To realize the core concept of the platform, it must be based on information integration and realized by processing a large number of terminal connection management data. The meaning of the smart older adults care service platform includes the business operation mode of the platform, which reflects how the platform creates wealth and gains income, and also includes the corresponding technical architecture. The main content is the overall architecture of the system, the main functional modules to realize the value demands of platform stakeholders, and the key technologies to support the operation of functional modules and realize value creation. The framework of smart older adults care service mode is shown in Figure 3.

**Figure 4:**
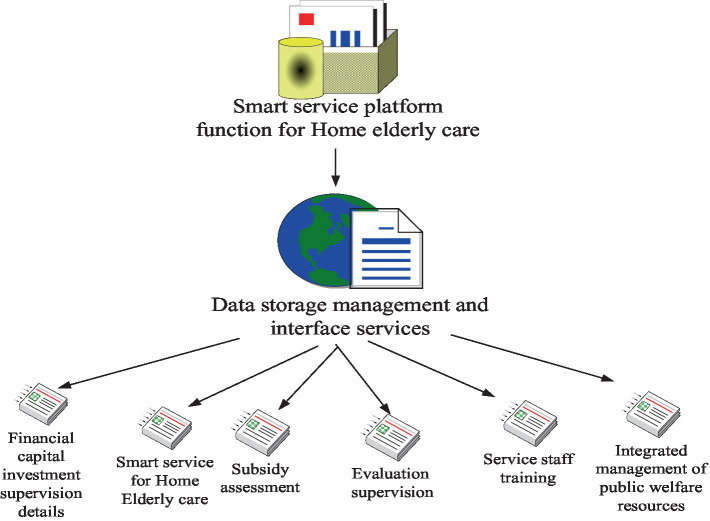
The functions of the wisdom service platform for the older adults.

The role of smart service platform for home-based older adults care mainly includes three aspects. First, all aspects of data and information collected are integrated and analyzed, and stored in a unified and standardized format for platform developers and customers to share. Second, the ability to purchase senior care services online is provided, enabling the older adults and service providers to complete service connection online. Third, the integration and optimization of older adults care service resources have been realized to assist the government in the management and supervision of the home-based older adults care industry. Therefore, the efficacy of the smart service platform for home-based older adults care mainly includes data information storage services, subsidy evaluation, evaluation and supervision, service personnel training, public welfare resource optimization and allocation management, etc. Details are shown in Figure 4.

Intelligent senior care service platform includes market platform features and technology platform features. The characteristics of the technology platform should be based on information integration, and scientific and reasonable system architecture is the basis for operating the market platform. The market characteristics of the smart older adults care service platform, as the intermediary between the supply and demand sides, actively respond and accurately match services to provide safe and reliable operation management services, which is the starting point for the smart older adults care service platform to realize its value. The establishment of smart senior care service platform is indispensable to the investment of government departments and social capital. Therefore, its goal in making decisions is to maximize the benefits. When the marginal revenue of the smart senior care service platform is equal to the marginal cost, its own net revenue can reach the maximum. The calculation formulas are:

(1) $$\frac{eW_{h}}{ex_{h}}=\frac{b_{h}\pi }{\sqrt[{2}]{\vartheta_{h}x_{0}+x_{h}}}$$

(2) $$\frac{eW_{m}}{ex_{m}}=\frac{\left(1-\pi \right)\left(1-\sigma \right)b_{m}}{\sqrt[{2}]{\vartheta_{m}x_{0}+x_{m}}}$$

The marginal cost is $A_{h}^{，}=e,A_{m}^{，}=1+\vartheta$ . Let $\frac{{\mathrm{eW}}_{i}}{{\mathrm{ex}}_{i}}=\frac{{\mathrm{eA}}_{i}}{{\mathrm{ex}}_{i}}\left(i=\mathrm{h,m}\right)$ , then the calculation formula is:

(3) $$\{\begin{array}{c} x_{h}={\left(\frac{b_{h}\pi }{2e}\right)}^{2}-\vartheta_{h}x_{0}\\ x_{m}={\left(\frac{\left(1-\pi \right)\left(1-\sigma \right)b_{m}}{2}\right)}^{2}-\vartheta_{m}x_{0} \end{array}$$

Assuming that the cost function is a function of the input level of the participants, the cost of the government and the cost of social capital are:

(4) $$B_{h}=e\left(\vartheta_{h}x_{0}+x_{h}\right)$$

(5) $$B_{m}=\left(1+\mu \right)\left(\vartheta_{m}x_{0}+x_{h}\right)$$

### 2.3. Problems in the smart older adults care service model

#### 2.3.1. Smart older adults care service resources are relatively scattered and service coverage is relatively limited

At this stage, although the smart senior care service is relatively convenient and efficient, the service resources are relatively scattered on the whole. Different types of information service platforms are unrelated to each other. A unified senior care service management system has not been built to integrate various types of senior care service resources to enhance the utilization rate of senior care service resources, thus avoiding the waste of resources (15). At the service provider level, the smart senior care infrastructure is still in the period of pursuing technological progressiveness. Most of the existing smart senior care equipment is built to integrate the development of real estate and industrial bases. It is committed to creating high-end luxury equipment or demonstration projects, which has led to some problems. The smart senior care service model driven and guided by grassroots health care has not formed a long-term cooperation mechanism with enterprises and non-profit organizations. The senior care service platform has a series of problems, such as no long-term cooperation mechanism with enterprises and non-profit organizations, limited equipment configuration and technical level, narrow coverage, insufficient promotion, and many other problems. Some high-quality intelligent older adults care equipment, such as intelligent older adults care apartment information system, only cover a part of residential communities and children’s welfare homes, and do not expand to a wider range. As a result, smart older adults care service is inconvenient for the older adults to obtain resources due to various factors such as scattered senior care service resources and limited service coverage, which increases the cost of senior care services.

#### 2.3.2. “Digital divide” impedes the dissemination of smart senior care services

The “digital divide” refers to the difference between the information rich and the information poor (16). Only when the older adults have crossed the “digital divide” can they get smart older adults care services in a real sense. At this stage, government departments have gradually opened various public service information to the public. However, if older persons do not have the ability to obtain such information, they are not able to obtain relevant welfare treatment or services. This is embodied in two aspects. On the one hand, from the viewpoint of the senior, the smart senior care service requirement is not a valid demand, but only a basic theoretical requirement. On the other hand, the older adult population is unique. For example, due to the values and frugal consumption concepts, as well as the lack of awareness of relevant departments, and the lack of strong publicity and guidance, many older adult people and children have doubts, fears and vague understanding of smart senior care services, which restricts the process of senior people mastering smart new technology products. At the same time, some older adult people are seriously affected by the traditional idea of “inheriting the family” and refuse to accept older adults care services. There are also some older adult people who think it is unhealthy to use high-tech to help themselves live, so they are more resistant to smart older adults care services.

### 2.4. Overview of the concept of primary health care institutions

**Figure 5:**
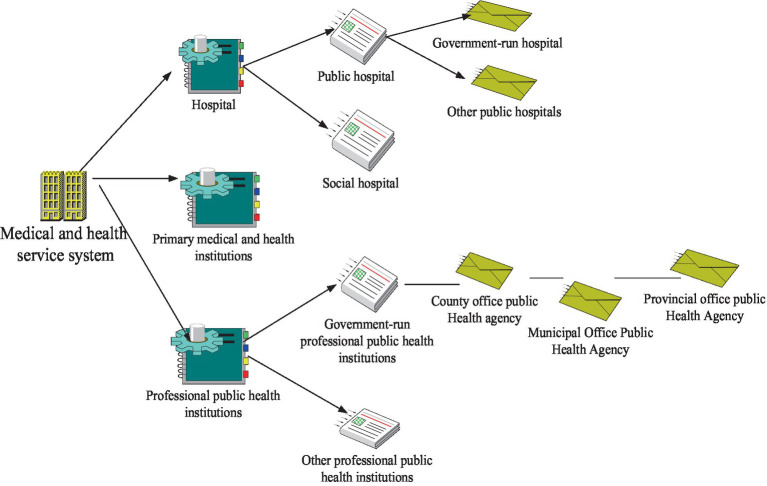
Schematic diagram of grassroots medical and health service system.

Medical and health resources refer to the social resources that people need to apply to carry out medical and health care activities. Broadly speaking, it refers to the general term of various economies of scale consumed or occupied by social development when enjoying medical and health services (17). Medical and health resources can be divided into many kinds. For example, medical and health resources can be divided into human resources (licensed (assistant) doctors, registered nurses, etc.), financial resources (health costs, government subsidies, etc.), and material resources (health institutions, institutional beds, medical equipment, etc.). In addition, it also includes some intangible resources, such as clinical medicine, medical technology, health management, environmental health policies, and regulations. Among them, the primary medical and health resources refer to the primary medical and health organizations, mainly including the medical and health resources possessed by various clinics. Primary health care organizations are community health centers and service points in large cities. Its primary task is disease prevention and control, health publicity and other public health management, as well as the treatment of some common diseases, which brings convenient and efficient basic medical services to urban residents. It can meet the most basic medical service needs of the masses, and is the core of the new round of medical reform. Primary medical and health organizations are committed to creating a “six in one” service mode and operation mode, that is, taking human health as the center, families as the unit, and streets as the basic scope, which integrates health education, prevention, health care, rehabilitation, family planning, and basic medical care. The schematic diagram of the primary health care delivery system is shown in Figure 5.

Basic medical services provided by primary medical and health institutions mainly include the following three aspects of knowledge. The first is general outpatient and emergency services, mainly including the treatment of common diseases, such as colds or chronic diseases. The second is basic public health management services. The third is proper referral advice, which is mainly for conditions that cannot be treated in primary care. According to the needs of patients, patients are transferred to appropriate designated medical institutions for diagnosis and treatment.

## 3. Experiment on the construction of intelligent older adults service model

The older adults in City A were investigated, mainly about their basic information and their requirements for smart older adults care services. Five hundred and twelve questionnaires were collected and 500 samples were actually collected. The sampling was mainly based on the survey of some older adult people in 5 districts of City A, and the survey results are representative. Table 1 shows the basic information of sampling.

**Table 1:** Basic information of sampling in City A.

Problem	Option	Number of people	100%
Gender	Male	240	48
	Female	260	52
Age	65–75	200	40
	76–85	200	40
	Over 86 years old	100	20
Monthly income	Below 3,500 yuan	200	40
	3,501–4,500 yuan	150	30
	4,501–5,500 yuan	100	20
	Above 5,501 yuan	50	10
Education level	Illiterate	220	44
	Junior high school education or below	240	48
	College degree or below	30	6
	Bachelor degree or above	10	2
Residence status	Living alone	110	22
	Live with children	100	20
	Live with a partner	220	44
	Nursing home	70	14
Take care of yourself	Cannot take care of yourself at all	80	16
	Can’ take care of yourself	300	60
	Can take care of yourself	120	24

As shown in Table 1, the proportion of men and women in the sample is similar, accounting for 48 and 52%, respectively. In terms of age, it is mainly between 65 and 85 years old, accounting for 80%. The older adults aged over 86 accounts for 20%. In terms of monthly income, most of the older adults selected were below 4,500 yuan, accounting for 70%, while those above 5,501 yuan accounted for the least, only 10%. According to the survey on the education level of the selected older adults, the older adults with junior high school education or below accounted for the largest proportion, 48%, while the older adults with bachelor’s degree or above accounted for the least, only 2%. Most of the older adults selected lived with their children or partners, accounting for 64%. The older adults living in nursing homes accounted for the least, only 14%. Most of the selected objects still have a certain self-care ability.

**Figure 6:**
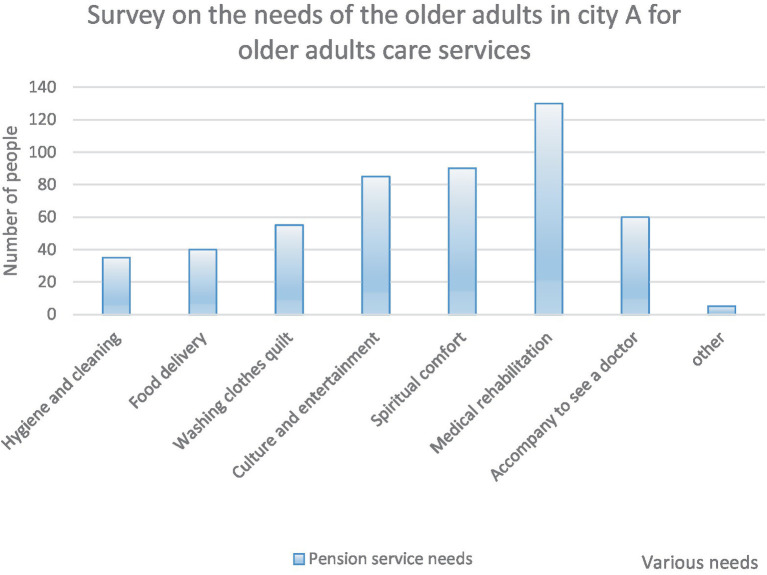
Survey on older adults care service demand in City A.

For the survey on what demands the extracted seniors have regarding smart senior care services, the specific findings are shown in Figure 6.

As shown in Figure 6, the older adults care service needs of the selected objects were mainly investigated in eight aspects. Among them, medical rehabilitation older adults were highly valued, and the number of people who chose was also the largest, with 130 people choosing, accounting for 26%. The need for cultural entertainment and spiritual solace is also highly valued by the older adults. The number of seniors who chose both was 175, accounting for a high 35%. In addition to the older adults who chose other options, the least number of people chose was cleaning. There were only 35 people chose, accounting for 7%.

**Figure 7:**
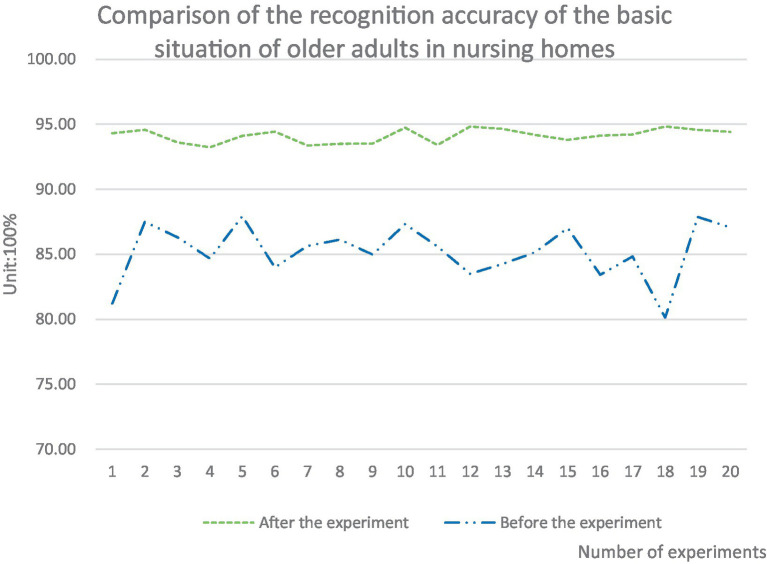
Comparison of recognition accuracy for the older adults in nursing homes before and after the experiment.

At this stage, despite the huge investment in senior care services, the utilization rate of older adults care service resources is not very high. There is even the phenomenon of overcapacity in some places for senior care services, with demand and supply for the senior citizens showing an unstable state. The drive for grassroots health care can integrate the older adults care service resources in various regions, which can help better understand the older adults care service models in various regions. The integration of various resources promotes the development of smart older adults care service model, which plays a role in promoting the growth of smart older adults care service in City A. In this paper, the intelligent older adults care service platform driven by primary health care was used to study the identification accuracy of the basic information of 100 older adult people. These data were tested 20 times. The experimental results were compared with those before the use of grassroots oriented health drive. After using the method studied in this paper for the experiment, the specific findings are presented in Figure 7.

As presented in Figure 7, the smart senior care service platform (that is, after the experiment) driven by grassroots health care was used. The recognition accuracy of the basic information of the selected 100 older adult people was much higher than that of the platform (that is. before the experiment) that was not built based on the grassroots medical and health drive. The accuracy of multiple experiments after the experiment was more stable than that before the experiment, and the fluctuation was controlled at about 1.59%. The fluctuation before the experiment was relatively large, and the fluctuation was controlled at about 7.76%. Among them, the accuracy after the 20 experiments was controlled above 93%, while the accuracy before the experiment was below 88%. Among them, the difference between the basic recognition accuracy of the older adults in the 18th experiment before and after the experiment was the largest, which was 14.7% higher than that before the experiment.

**Figure 8:**
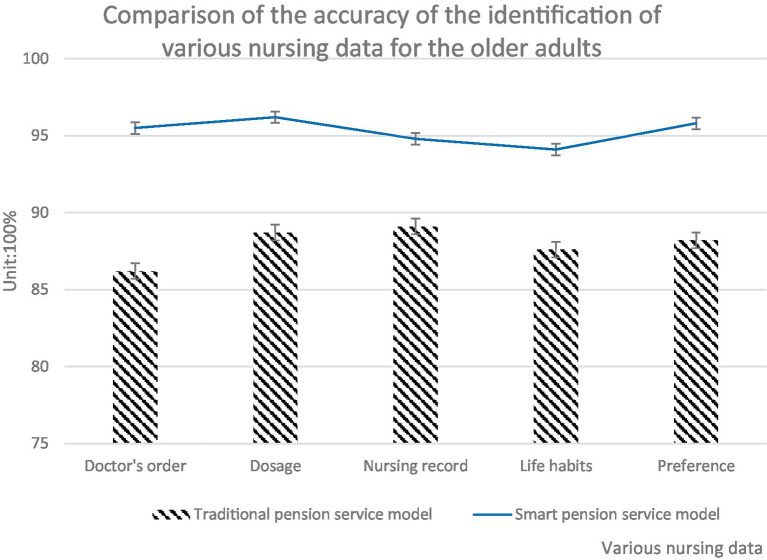
Comparison of the accuracy of the two service models in identifying various nursing data for the older adults.

The smart senior care service model promoted by primary health care was more intelligent than the traditional senior care service. It can improve personalized senior care services, and also help medical personnel to monitor the physical condition of the older adults in real time according to the intelligent service platform. Based on the smart senior care service model, it can accurately identify the medical advice, medication dosage, nursing records, living habits, and preferences of the older adults. Its recognition accuracy was higher than that of the traditional senior care service model. It can also better verify the medication status of the older adults, so as to reduce medical negligence and safety accidents. The specific comparison findings are illustrated in Figure 8.

As illustrated in Figure 8, the accuracy of intelligent senior care service mode in identifying various daily nursing data of the senior was higher than that of conventional senior care service mode. The identification accuracy of all kinds of daily nursing data of the smart senior care service model was above 94%, while that of the traditional senior care service model was below 90%. The intelligent senior care service model had the highest recognition accuracy in terms of drug dosage, 96.2%, which was 7.5% higher than the traditional senior care service model. The intelligent senior care service model had the lowest accuracy in life habits, only 94.1%, but was still 6.5% higher than the traditional senior care service model. The traditional senior care service model had the highest recognition accuracy in nursing records, 89.1%, but was 5.7% lower than the service model studied in this paper. The conventional senior care service model has the lowest recognition accuracy in medical advice, only 86.2, 9.3% lower than the service model studied in this paper. The post senior care service model driven by primary health care can record the daily care of the senior into an intelligent system. Doctors can search data at any time, and can further supervise the senior care home services, thus effectively improving the quality of care.

**Figure 9:**
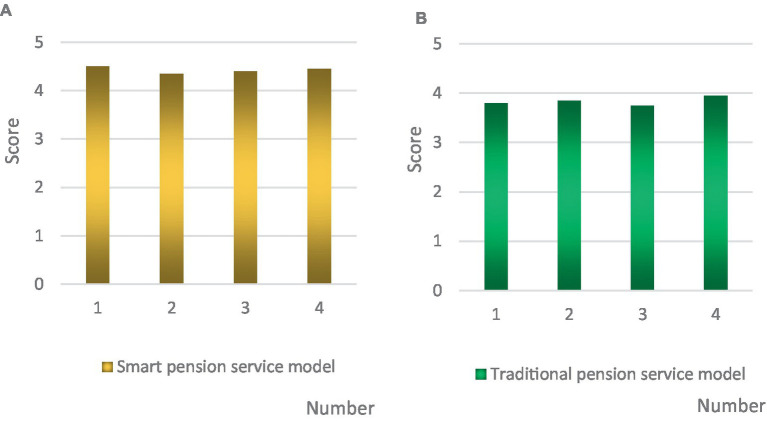
Comparison of two senior care service models. **(A)** Smart senior care service model. **(B)** Traditional senior care service model.

The smart senior care service model driven by primary health care has many advantages compared with the traditional senior care service model. An senior care home that is currently using the smart senior care service model in City A was randomly selected. Twenty staff members were selected from this senior care home to score and evaluate from the four aspects of effective integration of various service resources, realization of the senior multi service mechanism, promotion of the upgrading of senior care service products, and improvement of the construction of senior care professionals. These four aspects were numbered according to 1, 2, 3, and 4. The score was 1–5 points. The higher the score, the better the evaluation. The final result was the average of the scores given by 20 staff members, and the results were compared with the traditional service model. The specific comparison results are shown in Figure 9.

As shown in Figure 9, the staff selected by the smart senior care service model driven by grassroots health care were rated higher than the traditional senior care service model in all aspects. Among them, the score of smart senior care service mode was above 4.3, while that of traditional senior care service mode was below 4. Figures 9A,B were observed. It can be found that the smart senior care service model had the highest score in the number 1 of effective integration of various service resources, 4.5 points, which was 0.7 points higher than the traditional senior care service model. However, the smart senior care service model scored the lowest with respect to the implementation of multi service mechanism for the senior by number 2, only 4.35 points, but still 0.5 points higher than the traditional senior care service model. The traditional senior care service mode scored the highest in the aspect of number 4 to improve the construction of senior care professionals, 3.95 points, but still 0.5 points lower than the service mode studied in this paper. However, the lowest score of the traditional senior care service model was 3.75 points for number 3 to promote the upgrading of senior care service products, which was 0.65 points lower than the service model studied in this paper.

## 4. Conclusion

As the aging process of the population continues to increase, the needs of the older adults for senior care services are constantly rising. The wisdom older adults service model driven by primary health care is a combination of the state, social development, market, and other multi-participation and multi-faceted cooperation of the senior service system, the core of which is the construction on the wisdom older adults platform. Considering the needs of the older adults, intelligent goods have been developed, which not only need to meet the most basic needs of the older adults, but also pay attention to personalization. The advantages of primary medical care should be fully used to improve the requirements and the quality of senior care services. The senior care service industry chain has been continuously broadened to promote the healthy development of senior care services with grassroots medical care, and improve the participation and market competitiveness of the senior care market, thus creating a happy and healthy old age for the older adults. However, the research on smart older adults care services is a relatively cutting-edge topic, and the number of references is limited. In addition, the scientific research ability is not strong, and the research is not sufficient and detailed, so as to strive for further study in the future.

## Data availability statement

The original contributions presented in the study are included in the article/supplementary material, further inquiries can be directed to the corresponding author/s.

## Author contributions

All authors listed have made a substantial, direct, and intellectual contribution to the work and approved it for publication.

## Funding

The study was supported by Humanities and Social Sciences Research Project of the Ministry of Education: Research on the morphological evolution of health poverty and the optimization of governance path under the background of rural revitalization.

## Conflict of interest

The authors declare that the research was conducted in the absence of any commercial or financial relationships that could be construed as a potential conflict of interest.

## Publisher’s note

All claims expressed in this article are solely those of the authors and do not necessarily represent those of their affiliated organizations, or those of the publisher, the editors and the reviewers. Any product that may be evaluated in this article, or claim that may be made by its manufacturer, is not guaranteed or endorsed by the publisher.
