# Corrigendum: Construction of smart older adults care service model driven by primary health care

**DOI:** 10.3389/fpubh.2023.1319932

**Published:** 2023-10-25

**Authors:** Lechuan Zhang, Xiaoyan Xu

**Affiliations:** ^1^Shanghai Lixin University of Accounting and Finance, Shanghai, China; ^2^Shanghai Institute of Technology, Shanghai, China

**Keywords:** smart senior care service model, traditional older adults care service model, public health, primary medical and health care, pension service quality

In the published article, there was an error in affiliation(s) [1]. Instead of “Shanghai Lixin Institute of Accounting and Finance, Shanghai, China,” it should be “Shanghai Lixin University of Accounting and Finance, Shanghai, China.”

The authors apologize for this error and state that this does not change the scientific conclusions of the article in any way. The original article has been updated.

